# Observing ice structure of micron-sized vapor-deposited ice with an x-ray free-electron laser

**DOI:** 10.1063/4.0000185

**Published:** 2023-08-09

**Authors:** Seonmyeong Kim, Matlabjon Sattorov, Dongpyo Hong, Heon Kang, Jaehun Park, Jae Hyuk Lee, Rory Ma, Andrew V Martin, Carl Caleman, Jonas A Sellberg, Prasanta Kumar Datta, Sang Yoon Park, Gun-Sik Park

**Affiliations:** 1 Center for THz-Driven Biomedical Systems, Department of Physics and Astronomy, Institute of Applied Physics, College of Natural Sciences, Seoul National University, 08826 Seoul, Korea; 2 Center for Applied Electromagnetic Research, Advanced Institute of Convergence Technology, 16229 Suwon, Korea; 3 Department of Chemistry, The Research Institute of Basic Sciences, Seoul National University, 1 Gwanakro, 08826 Seoul, South Korea; 4 Pohang Accelerator Laboratory, POSTECH, Pohang 37673, Korea; 5 Photon Science Center, POSTECH, Pohang 37673, Korea; 6 School of Science, College of STEM, RMIT University, 124 La Trobe Street, VIC, 3000 Melbourne, Australia; 7 Department of Physics and Astronomy, Uppsala University, SE751 20 Uppsala, Sweden; 8 Center for Free Electron Laser Science CFEL, Deutsches Elektronen Synchrotron DESY, Notkestr. 85, 22607 Hamburg, Germany; 9 Department of Applied Physics, KTH Royal Institute of Technology, S106 91 Stockholm, Sweden; 10 Department of Physics, Indian Institute of Technology Kharagpur, 721302 West Bengal, India

## Abstract

The direct observation of the structure of micrometer-sized vapor-deposited ice is performed at Pohang Accelerator Laboratory x-ray free electron laser (PAL-XFEL). The formation of micrometer-sized ice crystals and their structure is important in various fields, including atmospheric science, cryobiology, and astrophysics, but understanding the structure of micrometer-sized ice crystals remains challenging due to the lack of direct observation. Using intense x-ray diffraction from PAL-XFEL, we could observe the structure of micrometer-sized vapor-deposited ice below 150 K with a thickness of 2–50 *μ*m grown in an ultrahigh vacuum chamber. The structure of the ice grown comprises cubic and hexagonal sequences that are randomly arranged to produce a stacking-disordered ice. We observed that ice with a high cubicity of more than 80% was transformed to partially oriented hexagonal ice when the thickness of the ice deposition grew beyond 5 *μ*m. This suggests that precise temperature control and clean deposition conditions allow *μ*m-thick ice films with high cubicity to be grown on hydrophilic Si_3_N_4_ membranes. The low influence of impurities could enable *in situ* diffraction experiments of ice nucleation and growth from interfacial layers to bulk ice.

## I. INTRODUCTION

The formation of ice is a complex and interesting process that plays a crucial role in various fields, ranging from microbiology^1,2^ to global climate.^3–5^ For example, it is known that water ice clouds are responsible for Earth's radiation balance by reflecting incident solar radiation and absorbing and re-emitting IR radiation from the surface of Earth. Recently, the growing threat of global warming has led to intense research interest in the feasibility of artificially modifying clouds to shift the Earth's radiation balance.

Understanding ice formation of ice crystals with a size in a micrometer range is especially important because they play an important role in the formation of clouds and precipitation.^6–9^ The size, shape, and facet orientation of ice crystals affect their ability to absorb and scatter sunlight, which in turn affects the Earth's radiation balance and climate.^10–12^ The micrometer-sized ice is of great interest in cryobiology as well.^1,2,13^ For example, the formation of ice crystals during freezing and thawing can damage cells, which is problematic for the preservation of cells and tissues for medical and research purposes.^14^ In outer space, micron-sized ice grains are abundant and considered to play a key role in the formation of planets and other celestial bodies.^15^ The ice grains can clump together to form larger particles, which eventually coalesce to form proto-planets.^16,17^

Despite the wide interest in research in various scientific disciplines, our understanding for the structure and nucleation mechanisms of micrometer-sized ice crystals is still very limited. This is partly due to the lack of appropriate experimental methods that can determine the structure of ice samples of these sizes with sufficiently high sensitivity. X-ray diffraction experiments have been used for determining the ice structure and provided valuable information about the stacking disorder structure of ices formed from supercooled liquid or vapor.^18–20^ These experiments have revealed that the ice structure is composed of randomly stacked hexagonal and cubic layers. However, because of the low scattering cross section of water ice for x-rays, the experimental observation has been limited to ice samples with the thickness of a few mm or greater.^21,22^ On the other hand, electron diffraction^23,24^ and scanning probe microscopic techniques, such as scanning tunneling microscopy (STM) and atomic force microscopy (AFM),^25^ can observe the structure and nucleation process of ice that occurs on the nanometer scale at the surface. These techniques have provided valuable insight into the understanding of ice nucleation in the initial stage. However, for ice crystals of intermediate sizes, i.e., in the *μ*m range, the direct measurement of the ice structure remains an experimental challenge.

To this end, we have utilized an x-ray free electron laser (XFEL) to observe the lattice structure of *μ*m-thick crystalline ice films grown by vapor deposition on a Si_3_N_4_ substrate surface at the temperature of 130–135 K in the ultra-high vacuum (UHV) condition. Due to the low scattering cross section of water ice, high-intensity x-ray sources, such as synchrotrons or XFELs, are required to efficiently capture diffraction data from *μ*m-thick ice films. The ability to focus the ultrashort XFEL pulses while maintaining photon flux increases intensity, improves signal-to-background ratio, and also allows captures of crystal structures in single-shot images, avoiding contamination of the ice samples from environmental gases during data accumulation.

We found that in the initial stage of the film growth, the structure of the ice sample was strongly influenced by the surface properties of the substrate, exhibiting a high degree of cubicity (I_*c*_ structure) on the hydrophilic Si_3_N_4_ surface. As the ice film grew thicker by vapor deposition, the cubicity gradually decreased and the hexagonal (I_*h*_) structure became prevalent in the film. The evolution from I_*c*_ to I_*h*_ structure appeared over the range of several *μ*m of film thickness, which is greater than the expected decay length on the order of nanometers for the effect of substrate surface on film growth.

## II. EXPERIMENTAL SETUP

**FIG. 1.**
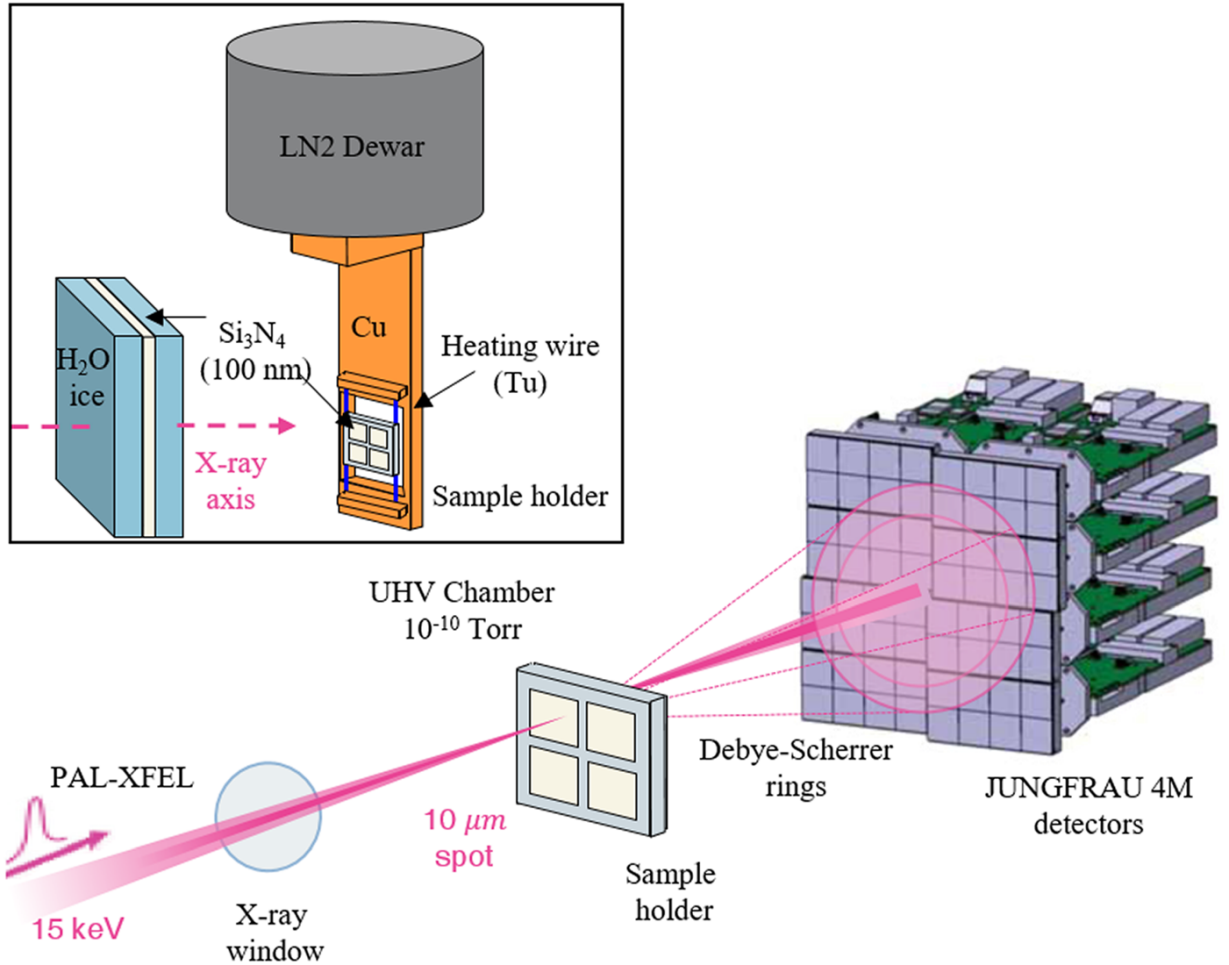
PAL-XFEL transmission x-ray diffraction test method using an ice deposition film formation system in an ultra-high vacuum chamber.

The experiments were performed in a custom-built UHV chamber at the PAL-XFEL XSS beamline. The UHV chamber was equipped with a sample holder and the facilities for ice film deposition and characterization, and it was connected through a gate valve with a diamond x-ray window to the XSS beamline. An intense x-ray pulse that passed through the window was diffracted from an ice film grown on the sample holder. The diffraction signals were measured as a Debye–Scherrer ring on a JUNGFRAU 4M detector installed on the opposite side of the chamber, as shown in Fig. 1.

### A. Vapor deposition ice in UHV chamber

A *μ*m-thick ice film was prepared by depositing water vapor on the surface of a Si_3_N_4_ membrane substrate mounted on a temperature-controllable sample holder. The sample holder structure is schematically drawn in Fig. 1. A cold head made of Cu was connected to a stainless steel dewar containing liquid nitrogen (LN_2_). A sample holder made of stainless steel was installed on the cold head through a pair of tungsten wires. The temperature of the sample holder was variable between 120 and 600 K by cryogenic cooling through a liquid nitrogen dewar and resistive heating of tungsten wires. The temperature measurement and control was achieved with accuracy less than 0.1 K using a K-type thermocouple attached to the sample holder and a Eurotherm 3504 programmable temperature controller.

The sample holder had a silver behenate (AgBH) calibration sample for x-ray diffraction image correction and a Si_3_N_4_ membrane as a substrate for ice deposition. Si_3_N_4_ membranes are commonly used for x-ray windows because of their excellent properties, such as high mechanical strength, uniformity, thermal stability, and high x-ray transmittance.^26^ Si_3_N_4_ substrates with hydrophilic oxygen termination were purchased from NORCADA. The Si_3_N_4_ membrane had a square shape with a size of 10 × 10 mm^2^ and a thickness of 100 nm, and it was supported by a Si frame with a size of 15 × 15 mm^2^ and a thickness of 500 *μ*m. The surface of the Si frame was coated with 400 nm thick Cr/Cu for efficient heat conduction to the sample holder. The surface of the Si_3_N_4_ membrane was treated with an oxide-based hydrophilic coating of oxygen rich silicon oxynitride (SiNxOy) with a thickness less than 10 nm for efficient growth of ice film on the surface. The hydrophilicity of the oxygen-terminated Si_3_N_4_ substrate was confirmed by contact angle measurement, which revealed the angle of about 25°. The Si_3_N_4_ substrate surface was cleaned by annealing up to 600 K inside a UHV chamber to remove surface contaminants.

Experiments for ice film preparation and characterization were conducted in the UHV environment to prevent the deposition of impurities, such as hydrocarbons, that affect the ice nucleation process. Also, the deposition of additional water vapor on the sample surface was minimized during x-ray diffraction experiments. The UHV chamber was evacuated by a 250 l/s turbomolecular pump to a base pressure better than 1 × 10^−9^ Torr at 300 K. During the sample cooling, the dewar functioned as a cryopump to lower the base pressure below 2 × 10^−10^ Torr. The mass spectra measured with a residual gas analyzer confirmed that the gas composition inside the UHV chamber consisted only of hydrogen (m/z = 1 and 2).

An ice film with a thickness up to 50 *μ*m was deposited on the Si_3_N_4_ membrane by back-filling the entire UHV chamber with water vapor through a precision leak valve. Ultra-pure de-ionized water (Milli-Q) with electrical resistivity of 18.2 MΩ cm and organic carbon content less than 3 ppb was stored in a glass vial rinsed with ultra-pure H_2_O. The water in a vial was further purified by repeating freeze-pump-thaw cycles prior to injection into the UHV chamber. A few cycles of vapor injection to the chamber and evacuation were performed prior to the experiment so as to remove weakly bound impurity molecules from the surfaces of injection pipes and chamber wall and to replace them with H_2_O. After this procedure, only the mass peaks due to H_2_O (m/z = 16–18) could be identified, indicating that water vapor consisted more than 99.8% of the residual gas composition. Next, an ice film was prepared in the following procedure. The chamber was evacuated to a UHV base pressure, and the sample holder was cooled by liquid N_2_. The cooling rate was controlled less than 1 K/s by the proportional integral differential (PID) control of resistive heating to prevent thermal stress, which may crack a Si_3_N_4_ membrane. When the sample was cooled to the desired deposition temperature of 130 K, H_2_O vapor was introduced into the chamber and deposited on the hydrophilic Si_3_N_4_ membrane surface. An ionization gauge monitored the water vapor pressure during the deposition. The thickness of an ice film and its growth rate were controlled by adjusting the water vapor pressure and the exposure time. The film thickness was also estimated by conducting temperature programmed desorption experiments for separate samples using the residual gas analyzer as a detector. The details of the temperature-programmed desorption method have been described elsewhere.^27–29^ After the ice film preparation, the precision leak valve was closed and the chamber was evacuated to reach the base pressure in less than one minute. Then, the gate valve to the detector was opened to start the x-ray diffraction experiment.

### B. X-ray diffraction experiments on deposited ice

All the x-ray diffraction experiments were performed at the XSS beamline of PAL-XFEL in transmission geometry. An x-ray pulse with a photon energy of 15 keV and a pulse duration of 50 fs passes through a diamond window in the center of the gate valve, connecting the PAL-XSS beamline and the UHV chamber, and is focused onto the sample surface with a focal size of 10 *μ*m.

X-ray diffraction experiments on the *μ*m-thick vapor deposited ice film were performed with a fluence of 177.41 J/cm^2^ and an average exposure of 100 shots, which was confirmed as a viable condition for accurately measuring the ice structure without radiation damage. The observed x-ray diffraction images were calibrated and analyzed as follows. AgBH reference powders with well-defined diffraction peak structures were measured on the same plane to locate the center and calibrate the pixel position of the x-ray diffraction image. The momentum transfer *q* of each pixel was then calculated according to *q* = 4*π*/*λ* sin(*θ*), where *λ* is the x-ray wavelength, and 2*θ* is the scattering angle, from which each diffraction pattern was converted to a radial intensity profile that was solid-angle and polarization corrected. Supplementary Fig. 1 shows that from the diffraction pattern of AgBH and the distance from the sample to the detector of 32.1 cm, the center of the diffraction image observed at the detector is corrected at the expected peak positions of AgBH. In addition, in order to eliminate the x-ray diffraction background caused by the sample holder and other objects assembled in the UHV chamber, the background shot was measured with only the Si_3_N_4_ membrane under the same conditions to obtain a diffraction image without an ice film from which the background was removed. Additional analytical details can be found in the supplementary material.

## III. RESULTS AND DISCUSSION

The focused x-ray pulse had a pulse energy of 0.6 mJ, so the corresponding fluence is 529 J/cm^2^. Intense XFEL beams with such a strong fluence can damage the sample membrane Si_3_N_4_ and *μ*m-thick ice films. In a previous study, when an XFEL pulse was irradiated on colloidal gold particles on a Si_3_N_4_ membrane, it was confirmed that gold nanoparticles with opposite charges were explosively evaporated by the photoelectric effect, creating holes in the Si_3_N_4_ membrane.^30^ In addition, it has been reported that the human HeLa S3 chromosome on Si_3_N_4_ irradiated by XFEL pulses damages not only the membrane but also the chromosome itself because the local temperature rise causes thermal expansion in a very short time due to the absorption of x-ray energy by the sample.^31^ When Si_3_N_4_ is exposed to XFEL, it can experience damage due to several processes, including photoionization, Auger decay, and electron-impact ionization.^32,33^ To minimize the damage caused by photoionization, hard x-rays of 15 keV are used. The photoionization cross section decreases with increasing x-ray energy, making it possible to suppress primary damage using harder x-rays.^34^ Furthermore, the low ionization cross section of silicon nitride and ice at 15 keV,^35^ along with the short pulse length of XFEL, might result in secondary damage caused by Auger decay and electron-impact ionization being negligible. This secondary damage mainly differs by the number of energetic electrons, which depend on the x-ray photons flux. Therefore, prior to x-ray diffraction experiments, it is essential to find an x-ray fluence condition for which there is no radiation damage to the membrane and to the ice deposited to the membrane. First, single-shot diffraction images were observed using x-ray fluences ranging from a very low transmission of 0.1% to an x-ray transmission of 33% to confirm the damage of the Si_3_N_4_ membrane on which 10 *μ*m thick ice was deposited. The attenuation of the x-ray pulse was varied with a Si wafer attenuator below the photon saturation limit of the detector.

**FIG. 2.**
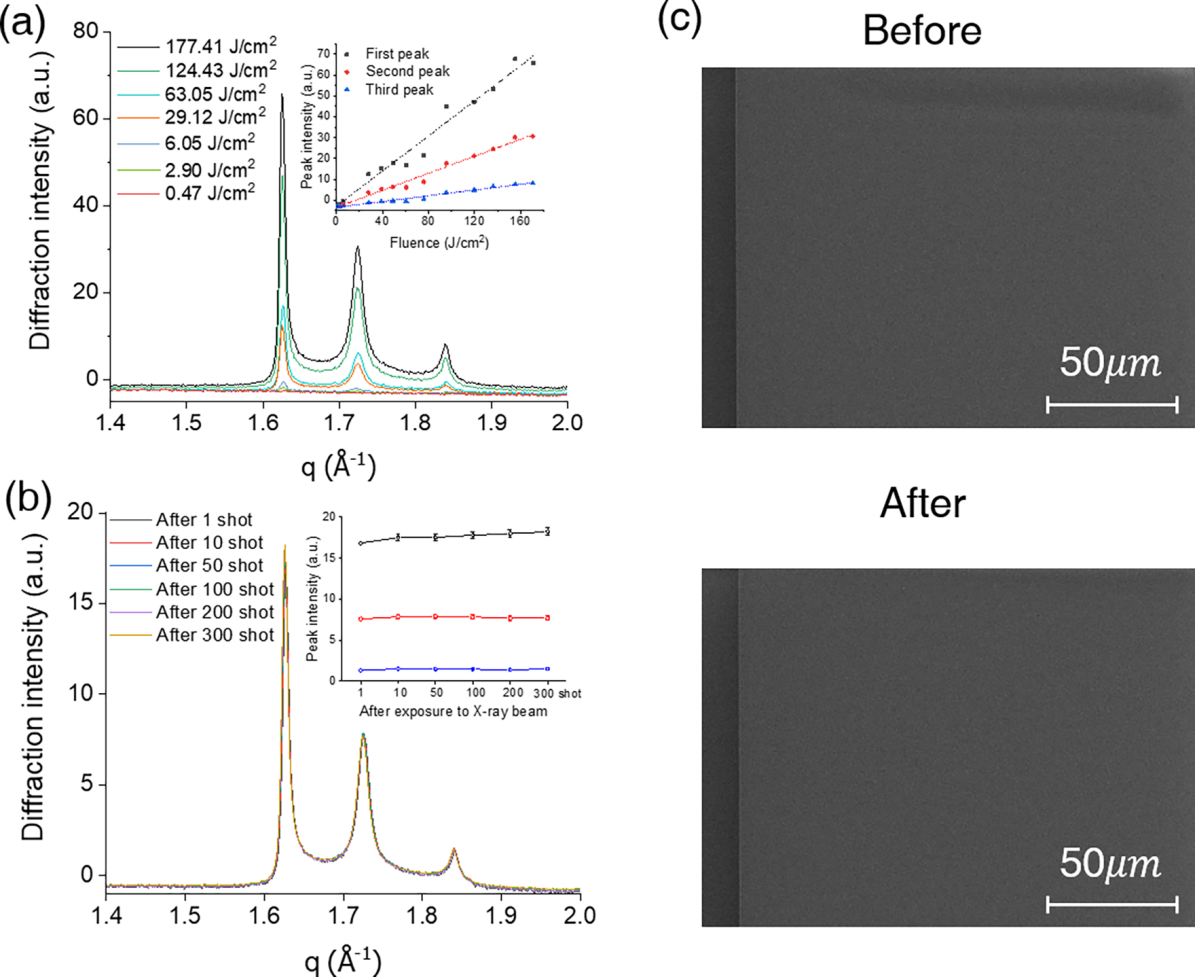
No radiation damage from intense x-rays to ∼10 *μ*m-thick ice films deposited on Si3N4 membranes. (a) Single-shot x-ray diffraction intensity using increasing x-ray fluence. (b) Single-shot diffraction intensity after x-ray exposure from single shot to 300 shots. The peak structure of ice crystal remains unchanged. (The peak intensity changes less than 5%, which is lower than 1 standard deviation.) (c) SEM image of Si3N4 surface before and after XFEL exposure.

In Fig. 2(a), the diffraction peaks due to the ice structures were poorly identified at very small x-ray fluences due to the low signal-to-noise ratio, which resulted in that the second and third peaks were buried below the background level. From 6.05 J/cm^2^ or higher, three peaks due to the ice structure were clearly observed. These three peaks coincide with the diffraction peaks of the hexagonal ice structure ([100], [002], [101]) and the cubic ice structure ([111]).^36^ In addition, when a diffraction pattern was obtained by focusing an x-ray pulse on the same location after irradiating a single x-ray, the same diffraction pattern was obtained continuously from 6.05 to 177.41 J/cm^2^. In the observed x-ray diffraction pattern, it can be confirmed that the diffraction peak intensity increases as the x-ray fluence increases [inset of Fig. 2(a)], but the ratio of the three diffraction peaks maintains a linear shape. This indicates that no radiation damage was observed in the ice structure. This result confirms that the structure of *μ*m-thick ice films can be measured by irradiating the sample with single-shot x-ray pulses with a maximum fluence of 177.41 J/cm^2^. In addition, it was confirmed that the Si_3_N_4_ film was not damaged after single-shot x-ray irradiation, since the TEM image of Si_3_N_4_ film did not show a discernible pattern, such as fracture or hole, due to radiation damage.

Figure 2(b) shows that there is no radiation damage to the Si_3_N_4_ film and the deposited ice film by exposure using an x-ray fluence of 177.41 J/cm^2^, where the diffraction pattern of the ice structure on the Si_3_N_4_ film can be observed by single-shot x-ray irradiation.

Comparing the x-ray diffraction pattern after single-shot exposure to 300 shots of exposure at the same position of ice film on the Si_3_N_4_ film, the observed ice structure showed no significant changes in the peak patterns. Furthermore, there is no evidence of cracks and hole formation by multiple x-ray irradiation. Inset figure shows that the peak intensities obtained remain the same within less than 5% variation.

This was also confirmed by scanning electron microscopy (SEM) and optical microscopy images. Figure 2(c) shows SEM images of Si_3_N_4_ before and after XFEL experiments. There were no cracks or holes due to strong x-ray irradiation in the SEM image.

**FIG. 3.**
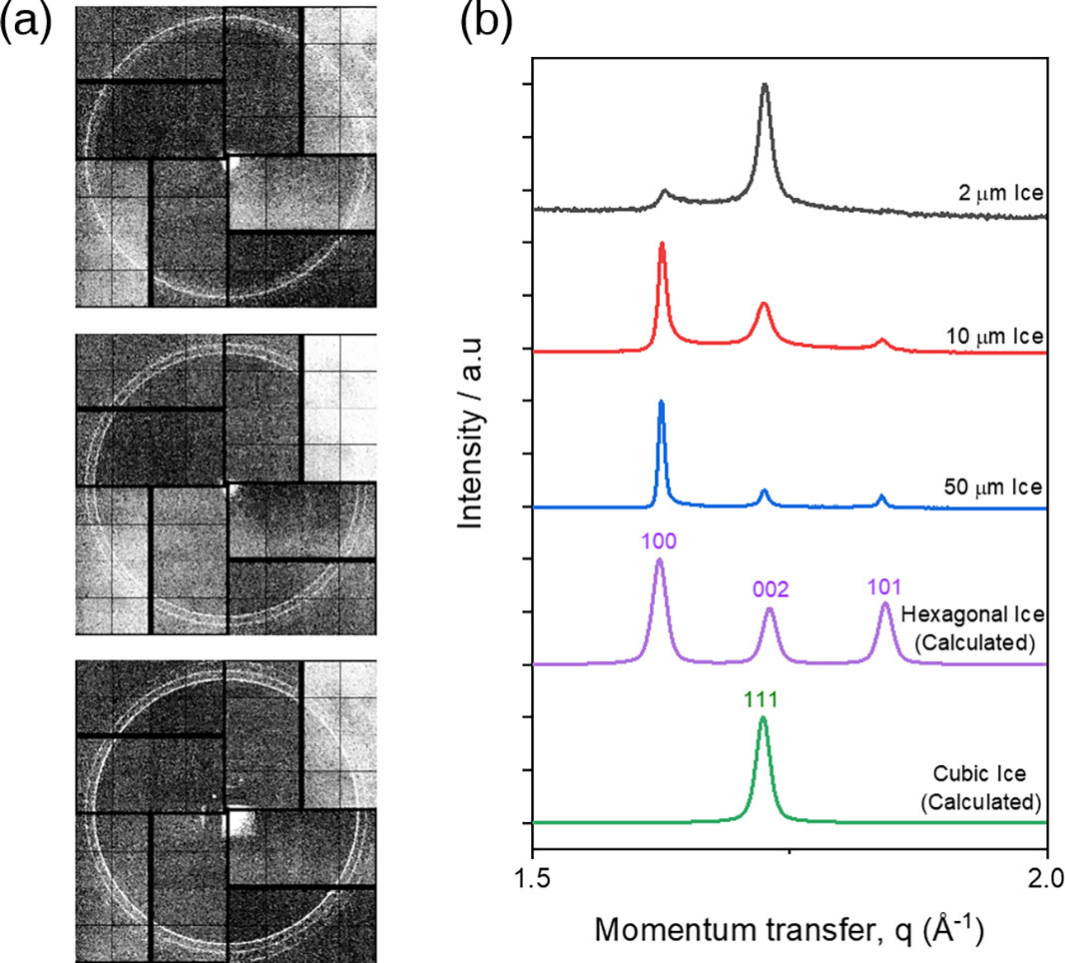
Observation of layered disordered ice structure of hydrophilic Si3N4 by x-ray diffraction of deposited ice. (a) Raw x-ray diffraction patterns from 2, 10, and 50 *μ*m-thick ice. (b) Processed x-ray diffraction intensity of vapor-deposited ice on Si_3_N_4_ at UHV conditions with different thicknesses is compared to calculated intensity profiles of hexagonal and cubic ice.

To determine the crystal structure of the *μ*m-thick ice films and their structural evolution during growth by vapor absorption, different thicknesses of ice were deposited on Si_3_N_4_ films at 130 K and the phases of each ice film were observed by x-ray diffraction. Figure 3 shows the results of an x-ray diffraction experiment on a *μ*m-thick ice film deposited at 130 K. At this deposition temperature, ice nucleates directly into the crystalline phase on the Si_3_N_4_ membrane substrate and there is no extensive amorphous diffraction signature visible around the momentum transfer from 1.5 to 2.0 Å^−1^.^12,36^

Figure 3(a) shows the appearance of three distinct ring patterns in the diffraction images of 2, 10, and 50 *μ*m-thick ice, and it can be seen that the three distinct peak intensities change for different thicknesses of ice. Figure 3(b) shows the averaged x-ray intensity profile for vapor-deposited ice films of 2, 10, and 50 *μ*m thickness. The experimental intensity profiles are compared with numerically calculated intensity profiles using DIFFaX^37^ of fully ordered hexagonal (I_*h*_) and cubic (I_*c*_) ice, which shows that the vapor-deposited, *μ*m-thick ice structure exhibits hexagonal structural characteristics with peaks at 1.62, 1.73, and 1.84 Å^−1^ ([100], [002], and [101], respectively). However, the relative peak intensities of the measured intensity profiles do not match the calculated perfectly ordered hexagonal ice structures. The ice structure we observed experimentally appears to have a diffraction intensity profile from a mixed phase with cubic ice that has a [111] peak at about 1.72 Å^−1^. The hexagonal diffraction patterns with [100], [002], and [101] around 1.62, 1.73, and 1.84 Å^−1^, respectively, are similar to the patterns discussed with ice I_*c*_-like structures as known as stacking-disordered ice.^12,36,38,39^

The diffraction feature of stacking-disordered ice and their structural evolution during growth by vapor deposition are shown in Fig. 3(b). The diffraction profile of the ice that have 2 *μ*m of thickness shows weak peak intensity at around 1.62 Å^−1^, which is the corresponding [100] peak of hexagonal ice, while the peak at around 1.84 Å^−1^, which is the corresponding [101] peak of hexagonal ice, is almost buried by the background signal. However, the second peak around 1.73 Å^−1^, which corresponds to [002] peak of hexagonal ice and the [111] peak of cubic ice, is dominating compared to the other two peak structures. This implies that the 2 *μ*m-thick ice film is composed of a large fraction of layers with cubic stacking sequences. The ice film began to decrease its cubic stacking sequences in the structure as the thickness increased further by vapor deposition. The relative intensity of peaks in the diffraction intensity profile observed at 10 and 50 *μ*m shows that the peak around 1.73 Å^−1^ including [111] peak from the cubic structure is decreasing, while peaks around 1.62 and 1.84 Å^−1^, including peaks only from hexagonal structures, are increasing as their thickness is growing. In particular, the peak around 1.62 Å^−1^ grows stronger than what is expected from the theoretical intensity profile of ice I_*h*_, indicating that the ice structure formed during vapor depositional growth has a preferred orientation along the [100] direction of hexagonal ice.

From the structural evolution observed in the diffraction experiments, it can be seen that the cubic fraction of stacking-disordered ice decreases as the thickness in- creases, which infers that the growth process of ice by vapor deposition may have a tendency to conform to the hexagonal structure despite having a thin template with high amount of cubic structure.

**FIG. 4.**
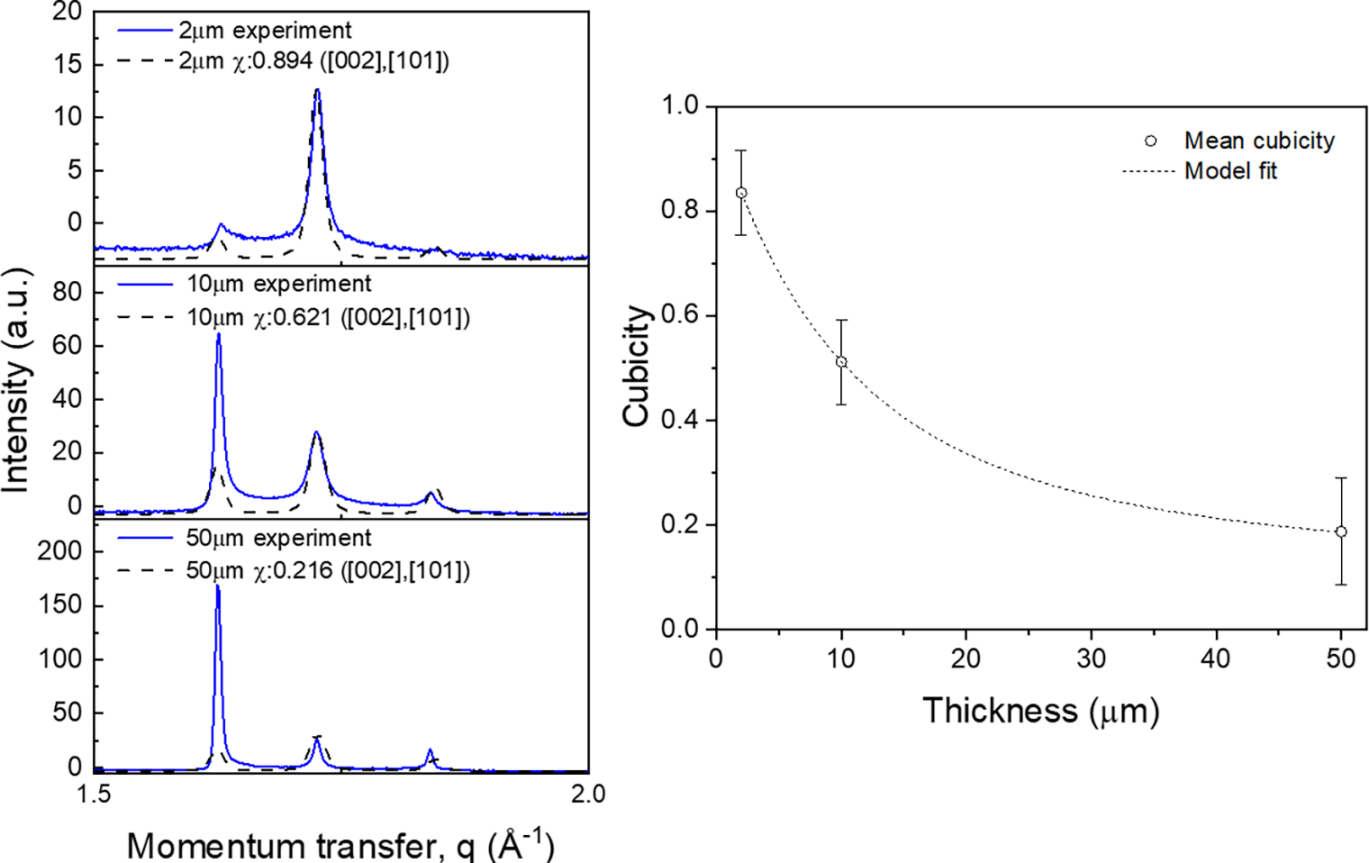
Stacking-disordered ice formation at the ice–vapor interface and decrease in cubic fraction during depositional growth to micrometer-sized ice. (a) Cubicity fraction calculated from the ratio between [002] and [101] peak. (b) Decrease in cubicity during depositional growth from 2 to 50 *μ*m with model fit.

The quantitative analysis of stacking disorder ice using DIFFaX^37^ is largely used to find a cubic/hexagonal layer fraction of stacking sequences. It is based on the assumption that, as the ice grows, a new layer of ice sequences is randomly determined between the hexagonal and cubic structure (i.e., the probabilities of new sequences switch from hexagonal to cubic and from cubic to hexagonal are independent of the presence of a certain sequence of layers beneath) However, our observed diffraction pattern showed the preferential orientation of ice in the [100] direction of the hexagonal ice structure. Previous studies also report that the growth of stacking disordered ice results in a distinct preferential orientation to the stacking plane parallel to the surface.^40,41^ Therefore, instead of obtaining a cubic fraction by comparing between experimentally observed diffraction profile and simulation prediction using DIFFaX, we obtain a cubic sequence fraction through comparison of the relative intensities of peaks around 1.62 Å^−1^ that contains the [111] peak of cubic ice structure and peaks around 1.84 Å^−1^ that only contains the [101] peak of hexagonal ice structure. Figure 4(a) shows the cubic sequence fraction (*χ*, cubicity) of 2, 10, and 50 *μ*m-thick ice. Comparing the peak near 1.62 Å^−1^ from the observed diffraction pattern (blue) to the prediction with stacking disorder and no preferential orientation (dashed black), it can be seen that the ice grows by vapor deposition with the preferential orientation in the hexagonal [100] direction. In addition, the cubic fraction decreased from 80% in 2 *μ*m-thick ice to 20% in 50 *μ*m-thick ice, implying that the ice structure progresses from a structure with high cubicity that includes a large fraction of cubic sequences to an ice structure with low cubicity with the majority of layers composed of hexagonal sequences when ice is grown through vapor deposition.

The observed structural progressions of *μ*m-thick ice can be interpreted as a hydrophilic property of the Si_3_N_4_ membrane affecting the ice nucleation process to initially have a preferred structure of cubic sequences. As the surface effect diminishes, the grown ice transforms into structures with hexagonal sequences due to screening by the water ice layers. In fact, new ice layer deposition in micrometer-thick ice films occurs at the ice–vapor interface, which is far from the hydrophilic Si_3_N_4_ membrane surface so the surface effect became negligible at the ice nucleating layers. Previous studies about the ice structure near hydrophilic and hydrophobic structures report that only a few interfacial layers of ice show the highly ordered cubic structure and it became the preferred hexagonal ice structure in the sub-nm to few nm range.^23,24^ Also, direct force measurements on a broad class of hydrophobic and hydrophilic surfaces show drastic exponential decay of hydrophilic and hydrophobic interaction energy, and surface effects are limited to only a few layers of water molecules, corresponding to a few nm.^42,43^

Another possible explanation of the structural evolution is that the temperature at the ice–vapor interface where new ice layer nucleation occurs gradually increases so that the hexagonal ice structure is favored. Many previous studies show that ice nucleation by vapor deposition prefers cubic sequences below 130 K and gradually transforms to prefer hexagonal sequences when temperature increases up to 180 K, because at low temperature ice does not have enough energy to transform from metastable cubic phase to thermodynamically stable hexagonal phase.^25,44^ Considering our sample holder structure and deposition condition, such as vapor pressure and temperature of the membrane surface, the temperature distribution across the micrometer-thick ice film and the ice–vapor surface temperature can be calculated by heat equation based on a thermodynamic model. The details can be found in the supplementary material.^46^ Based on the above-mentioned calculation, depositional temperature at the ice–vapor interface is the same at all ice films from 2 to 50 *μ*m, so the temperature effect on cubic-to-hexagonal structure evolution is negligible in this regard.

Many previous studies^13,18,45^ assumed that the stacking-disordered ice has a mechanism based on randomly switching sequences between cubic to hexagonal and hexagonal to cubic layer where there is correlation or memory in the stacking sequence. For example, the probability of forming a cubic ice layer on a cubic ice layer may be higher than that of forming hexagonal on cubic. It is difficult to transition back to a hexagonal sequence. Here, we only consider the correlation between nearest neighbor layers because an experiment showed that the correlation between the layers of next nearest neighbor has no substantial effect.^45^ To model the correlation between nearest neighbor layers, it is necessary to introduce two independent stacking probabilities, $\phi_{cc}$ and $\phi_{hc}$, which are the probabilities of forming cubic layer on top of a layer with cubic or hexagonal layer, respectively. Using the probabilities given above, the cubic ice fraction of the (*N* + 1)th ice layer, $\varphi_{N+1}$, formed on the top of *N*th ice layer can be expressed as

$$\varphi_{N+1}=\phi_{cc}\varphi_{N}+\phi_{hc}(1-\varphi_{N}).$$ (1)

The expression of $\varphi_{N}$ is given by

$$\varphi_{N}={\varphi_{0}k}^{N}+\left(k^{N}-1\right)\alpha ,$$ (2)

where $\varphi_{0}$ is the cubic ice fraction in the initial ice layer, $k=\phi_{cc}-\phi_{hc}$, and $\alpha =\frac{\phi_{hc}}{\phi_{cc}-\phi_{hc}-1}$.

In this study, we are interested in the average value of the cubic ice fraction, $\chi (N)=\frac{1}{N}\sum_{n=0}^{N-1}\;\varphi_{n}$, of a slab with thickness *D* = *N* Δd, where Δd is the thickness of a single layer. Then, χ(N) is written as

$$\chi (N)=\frac{1}{N}\left(\varphi_{0}+\alpha \right)\left[\frac{1-k^{N}}{1-k}\right]-\alpha .$$ (3)

Thus, the average cubic ice fraction for N layers depends on the ratio $(1-k^{N\;})/D$.

As shown in Fig. 4(b), the cubicity decreases, while the thickness increases by growing via vapor deposition, which is well explained by the model fit that considers growth of the ice film by vapor deposition based on stacking disorder. The fitted curve shows a characteristic decay length of cubicity of 10 *μ*m, which cannot be seen from previous experiment on nm-scale interfacial ice structure and mm-scale bulk water ice structure. This model could explain relatively long-range cubic-to-hexagonal structural evolution as the ice is initially nucleated into a highly ordered structure with high cubicity by a strong surface effect; although the surface effect is diminished in the nm range, the cubic structure gradually decreases through growing as stacking-disordered ice by vapor deposition.

Moreover, this finding implies that ice in extremely low impurity environments has long-term surface effects on formation and structural properties due to depositional growth at the water vapor interface. This unique crystallization process hints at a possible mechanistic pathway for the formation of ice I_*c*_ on hydrophilic surfaces in extraterrestrial environments.

## IV. SUMMARY AND OUTLOOK

In this study, we investigated the structure of micrometer-sized vapor-deposited ice films crystallized at low temperatures, relevant for cold clouds in the stratosphere and high troposphere, cryobiology, and protostars. The ice films have a stacking-disordered ice structure with large cubicity that decreases as the film thickness grows by vapor deposition. The crystalline structure of the *μ*m-sized ice films was observed by the help of intense x-ray diffraction experiment in transmission using the PAL-XFEL. Ice deposited on the Si_3_N_4_ membrane by vapor deposition under UHV conditions can be used to control the film thickness and avoid the influence of impurities on the nucleation process. This enables us to capture the structural transition from a highly cubic ice structure to a hexagonal ice structure during the growth mechanism, as new layers of ice are deposited on the *μ*m-scale when ice crystals are nucleated and grown by vapor deposition. Also, intense XFEL diffraction has enabled us to observe the structure of *μ*m-thick ice films for which relatively long-ranged cubicity decreases as the ice film grows. This cannot be observed by experiments that focus on interfacial ice structures using electron diffraction and bulk ice structures using reflection-type x-ray diffraction experiments. The single-shot x-ray diffraction experiments on *μ*m-thick ice films deposited on Si_3_N_4_ membrane show the possibility of the *in situ* type of diffraction experiments on ice nucleation and growth from the interfacial layers to bulk ice. While we have explored the structure of ice that is already grown as *μ*m-thick films, further studies will fully explore the structural evolution of ice from the initial nucleation stages to growth process by vapor uptake, which could form under clouds at high altitude or in deep space. In particular, it would be valuable to study ice grown from different initial states and conditions, by varying, for example, the surface and temperature. In addition, studies using specific surfaces that promote or prevent ice nucleation could elucidate how to control the structure of ice nucleation in different environments and can be used to model the global climate in depth.

## Data Availability

The data that support the findings of this study are available from the corresponding author upon reasonable request.
